# Preparation of biologically active *Arabidopsis* ribosomes and comparison with yeast ribosomes for binding to a tRNA-mimic that enhances translation of plant plus-strand RNA viruses

**DOI:** 10.3389/fpls.2013.00271

**Published:** 2013-07-22

**Authors:** Vera A. Stupina, Anne E. Simon

**Affiliations:** Department of Cell Biology and Molecular Genetics, University of Maryland, College ParkMD, USA

**Keywords:** plant ribosomes, TCV, 3′CITE, TSS, *Arabidopsis thaliana* protoplasts, virus translation

## Abstract

Isolation of biologically active cell components from multicellular eukaryotic organisms often poses difficult challenges such as low yields and inability to retain the integrity and functionality of the purified compound. We previously identified a cap-independent translation enhancer (3′CITE) in the 3′UTR of* Turnip crinkle virus* (TCV) that structurally mimics a tRNA and binds to yeast 80S ribosomes and 60S subunits in the P-site. Yeast ribosomes were used for these studies due to the lack of methods for isolation of plant ribosomes with high yields and integrity. To carry out studies with more natural components, a simple and efficient procedure has been developed for the isolation of large quantities of high quality ribosomes and ribosomal subunits from *Arabidopsis thaliana* protoplasts prepared from seed-derived callus tissue. Attempts to isolate high quality ribosomes from wheat germ, bean sprouts, and evacuolated protoplasts were unsuccessful. Addition of purified *Arabidopsis* 80S plant ribosomes to ribosome-depleted wheat germ lysates resulted in a greater than 1200-fold enhancement in *in vitro* translation of a luciferase reporter construct. The TCV 3′CITE bound to ribosomes with a three to sevenfold higher efficiency when using plant 80S ribosomes compared with yeast ribosomes, indicating that this viral translational enhancer is adapted to interact more efficiently with host plant ribosomes.

## INTRODUCTION

Studies of translation initiation using plant positive, single-stranded RNA viruses that lack a 5′ 7-methyl guanosine cap have revealed a wide range of mechanisms centered on highly structured, 3′ proximal cap-independent translation enhancers (3′CITEs) that bind to various host translation initiation factors (Simon and Miller, 2013). The 3′CITE of *Turnip crinkle virus* (TCV) is located in the 3′UTR and adopts an internal T-shaped structure (TSS) that topologically mimics a tRNA (McCormack et al., 2008) The TSS, formed from three hairpins and two pseudoknots, was shown to directly associate with yeast 80S ribosomes and 60S ribosomal subunits with a binding preference for the P-site (Stupina et al., 2008). Yeast ribosomes were chosen for these initial studies due to the availability of simple, well-established, highly efficient purification methods (Meskauskas et al., 2005; Stupina et al., 2008; Leshin et al., 2011). In contrast, methods available for plant polysome preparations are complex and result in limited yields (Lax et al., 1986; Mustroph et al., 2009, 2013). Although eukaryotic ribosomal complexes are highly conserved, differences exist in the structure of yeast and plant ribosomes and in the composition of translation initiation factors (Malys and McCarthy, 2011). Due to these differences, binding kinetics and other biochemical analyses using yeast ribosomes and the TSS were cautiously interpreted.

Development of simple, efficient procedures for plant ribosome preparation must account for the large central vacuole in mature plant cells (up to 90% of the cell volume; Hara-Nishimura and Hatsugai, 2011), whose contents can cause significant degradation of ribosomes during lengthy purification procedures. The pH environment of the plant vacuole is acidic, and its contents are enriched with proteases and RNases. For example, plant RNS2, a ribonuclease that participates in the normal decay of rRNA, uses the vacuole as the final destination for rRNA degradation (MacIntosh and Bassham, 2011). In the absence of commercially available plant RNase inhibitors, procedures that are appropriate for yeast ribosome isolation must therefore be modified to reflect conditions that are specific to plant cells.

In this report, we describe a simple, efficient method for isolation of plant ribosomes and ribosomal subunits with high yield and quality from *Arabidopsis thaliana* protoplasts prepared from seed-derived callus tissue. Purified, salt-washed (sw) *Arabidopsis* ribosomes complemented ribosome-depleted wheat germ lysates (WGLs) and enhanced translation of a luciferase reporter construct by 1200-fold, indicating high integrity and viability of the isolated ribosomes. Filter-binding assays demonstrated that a significantly higher percentage of purified *Arabidopsis* ribosomes associated with the TCV TSS compared with yeast ribosomes. These results indicate that the TCV TSS has evolved to maximize association with plant translation factors.

## MATERIALS AND METHODS

### PREPARATION OF SALT-WASHED 80S RIBOSOMES FROM PLANTS

For isolation of ribosomes from wheat germ (Kretschmer) and bean sprouts (fresh supermarket purchase), one volume of plant tissue was ground to powder in liquid nitrogen, resuspended in 5 volumes of plant buffer A [250 mM sucrose, 200 mM Tris–HCl pH 8.8, 30 mM MgCl_2_, 50 mM KCl, 1 mM DTT, and 1 mg/ml heparin], and cells lysed by incubation on ice for 5 min. Cellular debris was removed by centrifuging the lysates in a microcentrifuge at 11,000 rpm (10,000 × *g*) for 1 min at 4°C. For ribosome isolation from *Arabidopsis thaliana* protoplasts, sterilized seeds were plated on modified MS agar plates and grown into callus clumps as previously described in detail (McCormack and Simon, 2005). Approximately 1–2 ml of protoplasts and 3000–5000 pmol of ribosomes were obtained from 10 ml of packed callus clumps. Freshly prepared protoplasts (McCormack and Simon, 2005) free from any remaining buffer were either frozen at -80°C for storage of up to a year or directly used for ribosomes isolation. To generate evacuolated protoplasts, freshly prepared *Arabidopsis* protoplasts were subjected to ultracentrifugation through a 70–40% stepwise Percoll density gradient at 9,000 rpm (10,000 × *g*) for 1 h at 25°C using an SW 40 Ti rotor to separate cells into fractions with and without vacuoles (Komoda et al., 2004). One volume of packed protoplasts or evacuolated protoplasts was gently resuspended in ice-cold two to five volumes of plant buffer A and left on ice for 5 min to complete cell lysis. Cellular debris were removed by centrifuging lysates in a microcentrifuge at 11,000 rpm for 1 min at 4°C. To pellet 80S ribosomes, supernatants were pooled and subjected to ultracentrifugation by layering 2.5 ml of the supernatant over 2.5 ml of plant cushion buffer [25% glycerol, 500 mM Tris–HCl pH 8.8, 10 mM MgCl_2_, 50 mM KCl, 1 mM DTT, and 1 mg/ml heparin] in 5 ml polyallomer centrifuge tubes (13 mm × 51 mm, Beckman) followed by centrifugation at 4°C for 3 h at 50,000 rpm (268,000 × *g*) using an MSL-50 rotor (Beckman). Alternatively, 6 ml of supernatant were layered over 6 ml of plant cushion buffer in 13 ml polyallomer centrifuge tubes (14 mm × 95 mm, Beckman) and centrifuged at 4°C for 20 h at 28,000 rpm (139,000 × *g*) using a SW-40 rotor (Beckman). Ribosome pellets were gently rinsed with storage buffer [25% glycerol, 50 mM HEPES–KOH pH 7.6, 5 mM Mg(CH_3_COO)_2_, 50 mM NH_4_Cl, 1 mM DTT] and resuspended in the same buffer at a concentration of 2–7 pmol/ml. Ribosome concentrations were based on OD readings at A260, where A260 = 1 is equivalent to 20 pmol of ribosomes (Meskauskas et al., 2005). At this stage, ribosome suspensions are referred to as “1st spin” ribosomes and can be stored at -80°C for up to one year.

To remove any remaining ribosome-associated tRNAs, *Arabidopsis* ribosomes were treated as previously described for preparation of sw yeast ribosomes (Meskauskas et al., 2005). Specifically, 1st spin plant ribosomes were resuspended in 2.5 ml of buffer B [10% glycerol, 20 mM Tris–HCl pH 7.5, 5 mM Mg(CH_3_COO)_2_, 0.5 M KCl, 1 mg/ml heparin, and 1 mM DTT], GTP, and puromycin added to final concentrations of 1 mM, and the ribosome suspension incubated at 30°C for 30 min. After dilution to a final volume of 6 ml with buffer B, the solution was layered over 6 ml of buffer B cushion [25% glycerol, 20 mM Tris–HCl pH 7.5, 5 mM Mg(CH_3_COO)_2_, 0.5 M KCl, 1 mg/ml heparin, and 1 mM DTT] in a 13 ml polyallomer centrifuge tube and subjected to centrifugation at 4°C for 20 h at 28,000 rpm using an SW-40 rotor (Beckman). Salt-washed ribosome pellets were gently rinsed with storage buffer and resuspended in the same buffer at a concentration of 2–7 pmol/ml. Ribosomes have been stored at -80°C for up to a year.

### PREPARATION OF 40S AND 60S RIBOSOMAL SUBUNITS

40S and 60S ribosomal subunits were isolated by subjecting 1st spin ribosomes to sucrose gradient centrifugation essentially as previously described for isolation of yeast ribosomal subunits (Stupina et al., 2008). 1st spin ribosomes were resuspended in 1 ml buffer D [50 mM HEPES–KOH pH 7.6, 10 mM Mg(CH_3_COO)_2_, 0.5 M KCl, 1 mg/ml heparin, and 2 mM DTT]. GTP and puromycin were added to final concentrations of 1 mM and the ribosome suspension incubated at 30°C for 30 min to remove remaining tRNAs. The ribosome suspension was concentrated to 150 μl using Amicon Ultra (100 K) columns (Millipore) and applied to a 12.5 ml, 10–30% sucrose gradient prepared in buffer D and then subjected to centrifugation at 4°C for 18 h at 20,000 rpm (71,000 × *g*) in a swinging bucket SW-40 rotor (Beckman). After centrifugation, the top 1.5 ml of the gradient was discarded and the remaining material distributed into 0.5 ml fractions. Four microliters of each fraction was loaded onto a 1.2%, 0.5 × TBE/agarose gel for detection of 40S/60S subunits, and fractions containing the subunits combined into separate pools. The buffer was exchanged to 50 mM HEPES–KOH pH 7.6, 5 mM Mg(CH_3_COO)_2_, 50 mM NH_4_Cl, 10% glycerol, 1 mM DTT, and the suspension concentrated by subjecting each pool to several rounds of centrifugation through Amicon Ultra (100 K) columns. After glycerol was added to a final concentration of 25%, ribosomal subunits were stored at -80°C for up to a year.

### RIBOSOME STABILITY ASSAYS

Packed protoplasts (20 ml) were re-suspended in either 100 ml of yeast buffer A [10% glycerol, 20 mM HEPES–KOH pH 7.6, 5 mM Mg(CH_3_COO)_2_, 50 mM NH_4_Cl, 1 mM DTT, and 1 mg/ml heparin] or in 100 ml of plant buffer A [250 mM sucrose, 200 mM Tris–HCl pH 8.8, 30 mM MgCl_2_, 50 mM KCl, 1 mM DTT, and 1 mg/ml heparin] and suspensions incubated at 4°C and 10°C. Aliquots (25 ml) were collected at 1, 2, and 4 h time points and total RNA extracted by bringing the aliquot volume to 300 ml with water, addition of 300 ml of phenol/chloroform [1:1] and ethanol precipitating the aqueous fraction. Purified RNA products were then resolved on a 1.2%, 0.5 × TBE agarose gel.

### *IN VITRO* TRANSLATION USING RIBOSOME-DEPLETED WHEAT GERM LYSATES

Commercially purchased WGL (Promega) was added to ultra-clear centrifuge tubes (5 mm × 41 mm, Beckman) and centrifuged at 40,000 rpm for 5 h at 4°C using an MSL-50 rotor (Beckman). Supernatant containing depleted WGL was used for the *in vitro* translation assays. The ribosome pellet was re-suspended in storage buffer and analyzed by gel electrophoresis to approximate the amount of 80S ribosomes removed from the lysate (approximately 0.5 pmol of ribosomes were removed from each 10 μl of WGL). *In vitro* translation assays (10 ml reactions) were performed according to the manufacturer (Promega), using 1.4 pmol of luciferase reporter RNA. Two different reporters were tested: (1) uncapped firefly luciferase reporter RNA flanked by TCV 5′ UTR and 3′ sequences from position 3661 to the 3′ end at position 4054 (5′UTR-Fluc-3661); and (2) capped and polyadenylated renilla reporter RNA (Cap-Rluc-A). The 5′ cap was added to the 5′ terminus of the renilla luciferase reporter by supplementing the *in vitro* transcription reaction with m7G(5′)ppp(5′)G Cap analog (Ambion). The capped RNA was then polyadenylated with poly(A) Polymerase (New England Biolabs, NEB). To achieve 1× ribosome concentration, translation reactions were supplemented with 0.5 pmol of purified plant 80S ribosomes, or 40S and 60S ribosomal subunits. Translation assays were incubated at 25°C for 90 min and then placed on ice. Luciferase activity was determined using a single-reporter assay system (Promega) and a Modulus Microplate Multimode Reader (Turner BioSystems).

### FILTER-BINDING ASSAYS

Filter-binding assays were performed as previously described (Meskauskas et al., 2005). Ribosomes (15 pmol) and 1–60 pmol of [^32^P] 5′-end labeled TSS were combined in a total volume of 30 μl in binding buffer [80 mM Tris–HCl pH 7.4, 160 mM NH_4_Cl, 11 mM Mg(CH_3_COO)_2_, 6 mM β-mercaptoethanol, 0.4 mM GTP, 2 mM spermidine, 0.4 μg/ml of poly(U)]. Reactions were incubated for 30 min at 30°C, loaded onto pre-moistened Millipore HA filters (0.45 μ), and subjected to vacuum filtration. Each filter was washed three times with 3 ml of binding buffer and radioactivity quantified using a scintillation counter.

## RESULTS AND DISCUSSION

### *Arabidopsis* PROTOPLASTS AS A SOURCE FOR PREPARATION OF PLANT RIBOSOMES AND THEIR SUBUNITS

Analysis of associations between various types of molecules are often hindered by difficulties in purifying the molecules or molecular complexes. Until recently, yeast ribosomes have been used for studies on plant viral 3′CITEs that fold into tRNA-like structures because the biochemical properties of yeast ribosomes are well-characterized and the isolation procedures are relatively simple (Meskauskas et al., 2005; Leshin et al., 2011). Conducting such studies with more native materials requires isolation of plant ribosomes of good quality and in sufficient quantities, which is problematic due to the ease in which large vacuoles containing RNases and proteases are disrupted during the procedure.

To determine the most suitable plant source for isolation of high quality ribosomes, small-scale ribosome preparations were performed using wheat germ, bean sprouts, *Arabidopsis* protoplasts, and evacuolated *Arabidopsis* protoplasts, as described in the Section “Materials and Methods.” Since commercially available RNase inhibitors do not inhibit plant RNases, plant buffer A used to lyse plant material contained elevated pH (8.8) and a high concentration of Tris–HCl, conditions previously used for the isolation of plant polysomes (Davies and Larkins, 1973; Leaver and Dyer, 1974; Key et al., 1981). The quality of isolated ribosomes was determined by evaluating the integrity of ribosomal (r)RNAs *in situ* following separation of ribosomes by gel electro-phoresis.

**FIGURE 1:**
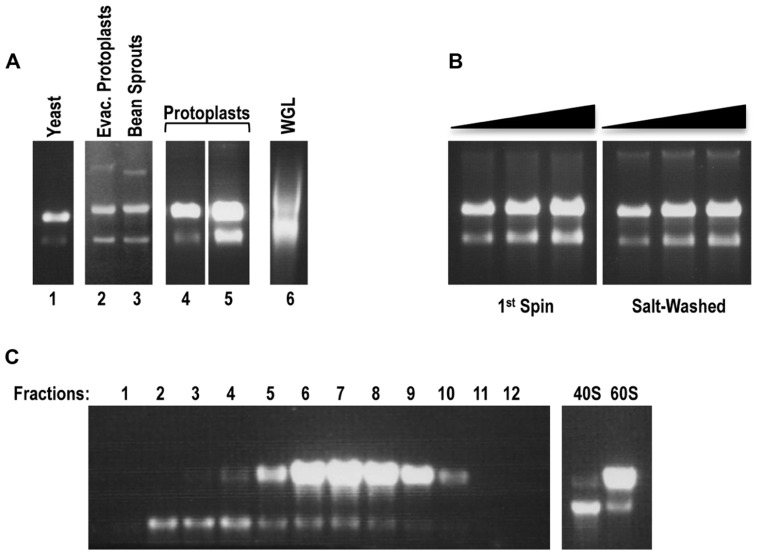
***Arabidopsis* protoplasts serve as the optimal source for the preparation of plant ribosomes and their subunits.** **(A)** *In situ* RNA gel electrophoresis analysis of small-scale ribosomal preparations (1st spin) from evacuolated *Arabidopsis* protoplast extracts (lane 2), bean sprouts (lane 3), *Arabidopsis* protoplasts (preparations 1 and 2, lanes 4 and 5). Yeast ribosomal preparation (lane 1) and aliquot of commercial WGL (Promega; lane 6) served as controls. Each preparation (1 ml, between 2.5 and 5 pmol of ribosomes) was resuspended in an equal volume of 100% formamide and the mixture directly loaded onto a 1.2%, 0.5×TBE agarose gel. rRNA was detected by staining gel with ethidium bromide. Note that although 80S ribosomes were loaded, the ribosome subunits disengage in the gel and thus the large and small rRNAs become separated. **(B)** *In situ* RNA gel electrophoresis analysis of the large-scale plant ribosomal preparation from *Arabidopsis* protoplasts: 80S 1st spin (left panel) and sw 80S (right panel). Three different concentrations (0.5, 0.75, 1 pmol) were loaded for each panel. **(C)** Sucrose-gradient separation of plant 40S and 60S ribosomal subunits. After centrifugation, the first 1.5 ml of the gradient were removed from the top of the tube and discarded. The remaining sucrose gradient containing subunits was subdivided into 0.5 ml fractions. Each fraction (4 ml) was resolved on a 1.2%, 0.5×TBE agarose gel for detection of rRNA from 40S and 60S subunits *in situ* (left panel). Fractions containing 40S and 60S subunits were combined into two pools. After buffer exchange and sample concentration, 2 μl of each pool were loaded onto the agarose gel to inspect the quality of the rRNA (right panel).

Non-salt-washed ribosome preparations, termed 1st spin ribosomes, were judged as good quality from all plant sources with the exception of wheat germ (**Figure 1A**), which did not produce ribosomes of sufficient abundance for detection using this procedure (data not shown). Interestingly, samples of commercial WGL that were directly applied to the gels contained ribosomes of poor quality and thus this material was not subjected to the 1st spin isolation procedure (**Figure 1A**). *Arabidopsis* protoplasts produced significantly higher yields of 1st spin ribosomes than bean sprouts and evacuolated protoplasts, and thus were chosen as the source for a large-scale preparation. Starting with 1–2 ml of packed protoplasts, 3000–5000 pmol of 1st spin ribosomes were isolated that were of equivalent quality as ribosomes isolated from small-scale preparations (**Figure 1B**, left panel). Ribosome preparations from both freshly prepared protoplasts and from protoplast pellets that were previously stored at -80°C were of equivalent quality and yield.

To remove residual tRNAs from 1st spin ribosomes, plant ribosomes were salt-washed using a method previously developed for yeast ribosomes (Meskauskas et al., 2005). Gel-electrophoretic analysis of sw plant ribosomes indicated that the ribosomes maintained their integrity (**Figure 1B**, right panel). Since subsequent purification procedures that start with poor quality 1st spin ribosomes can result in further degradation of the ribosomes, the lack of detectable degradation of these sw ribosome preparations indicates that the initial purification procedure effectively removed detrimental enzymes. Isolation of 40S and 60S subunits from 1st spin ribosomes was performed using a previously described procedure for isolation of yeast ribosomal subunits (Stupina et al., 2008). Gel electrophoresis of fractions containing 40S and 60S subunits displayed well-separated subunits that did not show any detectable degradation (**Figure 1C**). Fractions containing the individual subunits were combined and subjected to centrifugation through Amicon Ultra columns to exchange the buffer and to concentrate the subunits (**Figure 1C**, right panel).

### RNA STABILITY ASSAYS: PLANT BUFFER A PROVIDES A MORE STABLE ENVIRONMENT DURING RIBOSOMAL PURIFICATION COMPARED WITH YEAST BUFFER A

**FIGURE 2:**
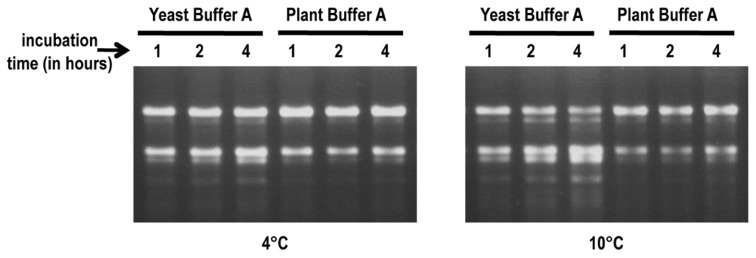
**RNA stability assays.** Frozen protoplast pellets were re-suspended in either yeast buffer A or plant buffer A. Suspensions were incubated at either 4°C (left panel) or 10°C (right panel). Aliquots were taken at 1, 2, and 4 h time points to extract rRNA. RNA (1 μg) was inspected for integrity on 1.2%, 0.5×TBE agarose gels.

RNA stability assays were conducted to determine if plant buffer A provides a more stable environment for plant ribosomes during the cell lysis step compared with the buffer used for yeast ribosome isolation (yeast buffer A; Meskauskas et al., 2005). Frozen protoplast pellets were resuspended in either yeast buffer A [10% glycerol, 20 mM HEPES–KOH pH 7.6, 5 mM Mg(CH_3_COO)_2_, 50 mM NH_4_Cl, 1 mM DTT, and 1 mg/ml heparin] or plant buffer A [250 mM sucrose, 200 mM Tris–HCl pH 8.8, 30 mM MgCl_2_, 50 mM KCl, 1 mM DTT, and 1 mg/ml heparin] and suspensions incubated for up to 4 h at 4 and 10°C. Total RNA isolated from samples removed at various time points were inspected for integrity on agarose gels (**Figure 2**). No visible degradation of the rRNA was observed after 4 h incubation at 4°C in plant buffer A and only minimal degradation was present at 10°C (**Figure 2**). In contrast, RNA cleavage products were visible after 4 h incubation in yeast buffer A at 4°C and degradation was significantly increased when the temperature was raised to 10°C. This suggests that the higher pH and elevated concentration of Tris–HCl in plant buffer A inhibits degradation enzymes and thus helps to maintain the integrity of the ribosomes during the isolation process.

### SALT-WASHED PLANT RIBOSOMES ARE FUNCTIONAL IN RIBOSOME-DEPLETED WGL FOR TRANSLATION OF A LUCIFERASE REPORTER mRNA

**FIGURE 3:**
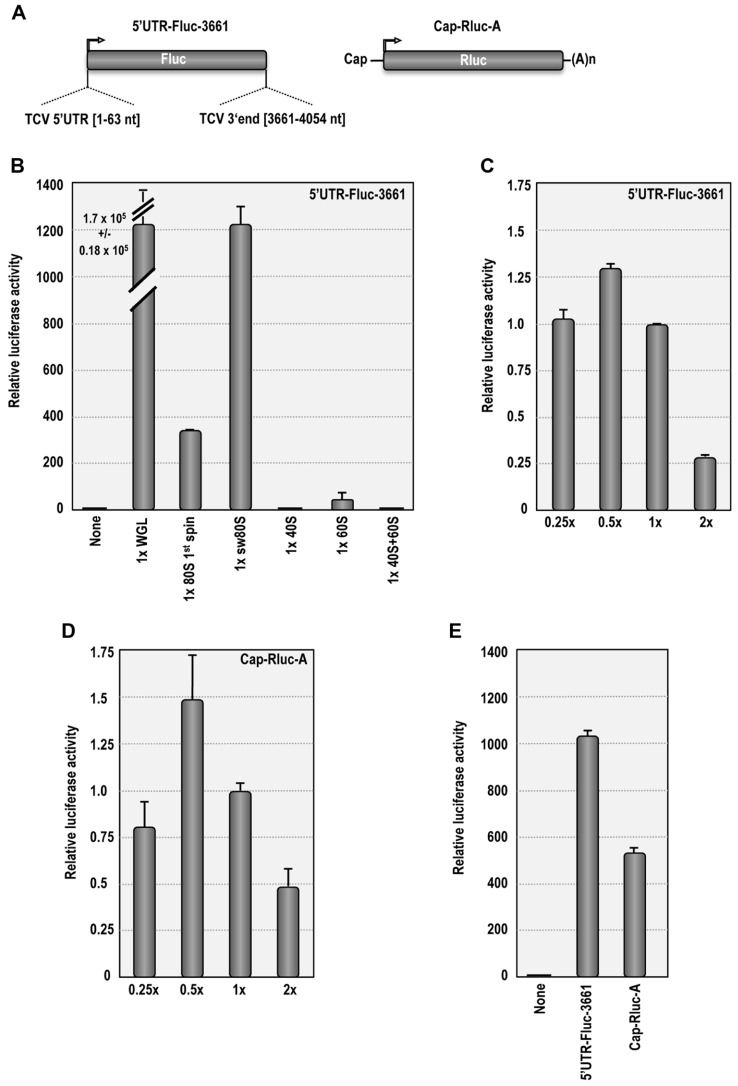
** Salt-washed plant ribosomes support cap-dependent and cap-independent translation in ribosome-depletedWGL.** **(A)** Map of 5′UTR-Fluc-3661 and Cap-Rluc-A reporter constructs. 5′UTR-Fluc-3661 contains the firefly luciferase ORF flanked by the TCV 5′UTR and positions 3661–4054. Cap-Rluc-A contains a capped, polyadenylated renilla luciferase ORF. **(B)** *In vitro* translation of 3′UTR-Fluc-3661 using ribosome-depleted WGL supplemented with either WGL ribosomes or *Arabidopsis* ribosomes or ribosomal subunits. None, no ribosomes added. **(C)** *In vitro* translation of 3′UTR-Fluc-3661 using ribosome-depleted WGL supplemented with different concentrations of sw *Arabidopsis* ribosomes. **(D)** Translation of Cap-Rluc-A using different concentrations of sw *Arabidopsis* ribosomes. **(E)** Comparison of cap-dependent (Cap-Rluc-A) and cap-independent (5′UTR-Fluc-3661) translation using 0.5× sw *Arabidopsis* ribosomes.

Purified plant ribosomes were tested for functionality in ribosome-depleted WGL (**Figure 3**) using luciferase reporter construct 5′UTR-Fluc-3661, which was previously used to detect synergy between the 5′UTR and 3′ region of TCV (Stupina et al., 2008; **Figure 3A**). In the absence of added ribosomes, translation using ribosome-depleted WGL remained at background levels (**Figure 3B**). When the reaction was reconstituted with ribosomes pelleted from WGL (~1 × concentration), translation of 5′UTR-Fluc-3661 increased by 1.7 × 10^5^-fold (**Figure 3B**). When 1st spin or sw *Arabidopsis* ribosomes were used at the same concentration to supplement the depleted WGL, translation of 5′UTR-Fluc-3661 increased by 340-fold or 1200-fold over background, respectively. Translation of 5′UTR-Fluc-3661 was maximal when sw ribosomes were added at a concentration of 0.5× (**Figure 3C**). The reduced level of translation resulting from supplemented *Arabidopsis* ribosomes compared with supplemented WGL ribosomes may have resulted from depletion of factors needed for translation when the WGL was subjected to ultracentrifugation to remove ribosomes. Alternatively, translation may be enhanced when both ribosomes and translation factors are derived from the same plant. Increased translation by *Arabidopsis* sw ribosomes over non-washed ribosomes suggests that salt-washing removes interfering tRNAs that might initially compete with the TCV TSS.

Supplementing ribosome-depleted WGL with 40S subunits (1×) did not result in detectable translation over background levels. Addition of 60S subunits (1×) stimulated translation by 40-fold over background levels, possibly due to residual 80S subunits (**Figure 3B**). Interestingly, addition of 40S and 60S subunits eliminated the 60S-induced translational stimulation, suggesting that purified 40S subunits compete with the residual 80S ribosomes during translation. It is not known why combined 40S and 60S subunits were unable to complement the ribosome-depleted extracts.

Salt-washed *Arabidopsis* ribosomes were also tested for their ability to support cap-dependent translation. Similar to 5′UTR-Fluc-3661, translation of Cap-Rluc-A reached maximal levels using 0.5× sw ribosomes, producing a 530-fold enhancement over background levels (**Figures 3D,E**). This result demonstrates that sw plant ribosomes are functional in ribosome-depleted WGL and can support both cap-dependent and cap-independent translation.

### SALT-WASHED PLANT 80S RIBOSOMES ASSOCIATE MORE EFFICIENTLY WITH VIRAL TSS COMPARED WITH YEAST 80S RIBOSOMES

**FIGURE 4:**
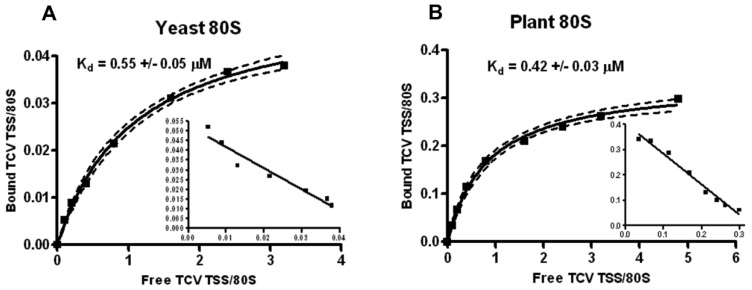
** Plant ribosomes associate with theTCVTSS at higher efficiency compared with yeast ribosomes.** One to 60 pmol of [^32^P] 5′-end labeled TSS were combined with 15 pmol of sw yeast ribosomes **(A)** or sw plant ribosomes **(B)**. *Y*-axis denotes the fraction of TSS bound per ribosome. *X*-axis indicates the ratio of input (free) TSS to ribosomes. Scatchard plots are included for each saturation curve. To generate a saturation curve and to calculate *K*_d_ values, each filter-binding experiment was conducted in triplicate.

The TCV TSS binds to 80S and 60S ribosomes and binding is important for translation (Stupina et al., 2008). Using filter-binding assays, the *K*_d_ for TSS binding to yeast 80S ribosomes was 0.45 mM, and binding was slightly enhanced for 60S subunits (0.34 mM). Binding to 40S subunits was non-specific (Stupina et al., 2008). To determine the *K*_d_ for TSS binding to *Arabidopsis* 80S ribosomes, similar filter-binding assays were conducted. The TCV TSS associated with yeast ribosomes with a *K*_d_ similar to that previously published (0.55 mM; **Figure 4A**), and plant ribosomes with a very similar *K*_d_ (0.42 mM; **Figure 4B**). However, only ~10% of yeast ribosomes interacted with TCV TSS, whereas at least 30% of the plant ribosomes associated with the TSS.

A recent report from our laboratory (Gao et al., 2012) provides additional analysis on the association of *Arabidopsis* sw ribosomes and their individual subunits with the TCV TSS and a second TSS located in the 3′UTR of *Pea enation mosaic virus* (PEMV). This PEMV TSS, unlike the TCV TSS, also participates in a long-distance kissing-loop (kl) interaction with 5′ proximal sequences and is therefore known as a kl-TSS. As previously reported for yeast ribosomal subunits (Stupina et al., 2008), the TCV TSS bound specifically to *Arabidopsis* 60S subunits (*K*_d_ = 0.23 mM) and non-specifically to *Arabidopsis* 40S subunits (Gao et al., 2012). Similar to the TCV TSS, the PEMV kl-TSS associated with plant ribosomes with a threefold enhanced efficiency compared with yeast ribosomes (Gao et al., 2012). Interestingly, unlike the TCV TSS, the PEMV kl-TSS interacted specifically with both 40S and 60S subunits from *Arabidopsis*.

In conclusion, we have developed a simple, efficient protocol for isolation of plant ribosomes that produces ribosomes of high yield and quality. Our results suggest that plant RNA viruses have evolved their translational *cis*-elements to maximize specific association with plant translation factors and that studies on cap-independent ribosome entry using plant mRNAs should use plant-derived ribosomes. We are hopeful that this method will be useful in further research into cap-independent translation mechanisms in plant viruses.

## Conflict of Interest Statement

The authors declare that the research was conducted in the absence of any commercial or financial relationships that could be construed as a potential conflict of interest.
